# A tutorial on Bayesian hypothesis testing of correlation coefficients using the BFpack-module in JASP

**DOI:** 10.3758/s13428-025-02846-5

**Published:** 2025-10-13

**Authors:** Joris Mulder, Julius Pfadt, Eric-Jan Wagenmakers

**Affiliations:** 1 Department of Methodology and Statistics, Tilburg University, Warandelaan 2, 5037 AB Tilburg, the Netherlands; 2 Department of Psychological Methods, University of Amsterdam, Amsterdam, The Netherlands

**Keywords:** Correlations coefficients, Hypothesis testing, Bayes factors, Posterior probabilities

## Abstract

Correlation coefficients play a central role in scientific research to quantify the (linear) association between certain key variables of interest. Currently, hypothesis testing of correlation coefficients, such as whether a correlation equals zero or whether two correlations are equal, is mainly done using classical *p* values, despite their known limitations. An important cause of this problem is the limited availability of statistical software that supports alternative, Bayesian testing procedures. To address this shortcoming, the current tutorial paper showcases how to perform Bayesian hypothesis tests on correlation coefficients using the new BFpack module in the free and open-source software program JASP. The module supports Bayesian tests of various types of correlations such as product–moment correlations, polyserial correlations, or tetrachoric correlations, among others. Partial correlations can be tested by controlling for certain covariates. Moreover, both dependent and independent correlations can be tested to be zero or tested against each other. This tutorial aims to get researchers acquainted with this new flexible testing paradigm, which avoids the limitations of classical methods, and to make the methodology widely available to the research community.

## Introduction

In the last decades, classical significance testing using Fisherian *p* values has been under severe scrutiny (Berger & Delampady, 1987; Sellke et al., 2001; Wagenmakers, 2007; Wasserstein & Lazar, 2016; Benjamin et al., 2018, among many others). Important arguments against its use include its inability to quantify evidence in favor of a null hypothesis (Rouder et al., 2009), its inability to distinguish between absence of evidence (i.e., low power) and evidence of absence (i.e., support in favor of the null) (Altman & Bland, 1995; Dienes, 2014), its inconsistent behavior if the null is true (i.e., even for extremely large samples there is still a strictly positive probability (namely the significance level) of incorrectly rejecting the null), its severe dependence on the (generally unknown) sampling plan of a researcher (Wagenmakers, 2007), and its inability to combine *p* values when testing multiple (one-sided) hypotheses (Braeken et al., 2015). Bayesian hypothesis testing using Bayes factors and posterior probabilities does not share these limitations. For this reason, there has been a huge development of Bayes factors for hypothesis testing in common research designs in applied (psychological) research such as *t* tests (Rouder et al., 2009), ANOVA designs (Klugkist et al., 2005; Rouder et al., 2012; Mulder & Gu, 2022), regression modeling (Rouder & Morey, 2012; Mulder & Olsson-Collentine, 2019), or structural equation modeling (Van Lissa et al., 2021), to name a few. Tutorial papers on the conceptual underpinnings are also available (Masson, 2011; Morey et al., 2016; Wagenmakers et al., 2018; Hoijtink et al., 2019).

**Table 1:** Types of measures of association depending on the measurement scale of the variables

	Scale of $Y_2$		
Scale of $Y_1$	Continuous interval	Polytomous ordinal	Dichotomous
Continuous interval	Product–moment	Polyserial	Biserial
Polytomous ordinal		Polychoric (I)	Polychoric (II)
Dichotomous			Tetrachoric

The current paper focuses on Bayesian testing of correlation coefficients. The test of whether a correlation is nonzero is ubiquitous in applied scientific research to assess whether there is a (linear) association between two variables. Other commonly observed tests include whether two dependent correlations are equal, which could either be overlapping (i.e., the correlations share a common variable) or nonoverlapping (i.e., the correlations do not share a common variable), or whether two independent correlations between variables measured on independent groups are equal. Moreover, depending on the measurement levels of the variables (either dichotomous, ordinal, or continuous) different types of correlation coefficients need to be considered. Table 1 gives an overview of the possible correlations for pairs of variables having different measurement scales.

Despite its well-known problems, classical significance testing using the *p* value is the most dominant methodology to test correlation coefficients. An important reason for this practice is the limited availability of statistical software that implements alternative approaches such as the Bayes factor, for testing correlation coefficients. For this reason, the R package BFpack (Mulder et al., 2021) was developed, which contains a broad collection of Bayes factor testing procedures, including a suite for default testing of different types of correlations using uniform priors. To facilitate the usability of this methodological framework, BFpack has been implemented as a module in JASP, an open-source statistical software program with a graphical user interface (JASP Team, 2024). This paper presents a tutorial on Bayesian testing of correlation coefficients using this module to provide researchers with a means to test scientific hypotheses on correlations in a statistically sound manner.

Although the JASP module allows users to test hypotheses with complex combinations of equality, order, or interval constraints (Mulder, 2016; Mulder & Gelissen, 2023), this tutorial focuses on the most common tests: whether a single correlation equals zero, whether two dependent correlations are equal, and whether two independent correlations are equal. Firstly, in order to illustrate the test of whether or not a single correlation equals zero, we consider a study from Mestek et al. (2008) where the interest was in understanding the association between physical activity (PA; measured objectively by a pedometer) and body composition (measured by the body mass index; BMI). By discretizing the variables in different categories (e.g., dichotomizing BMI results in ‘obese’ or ‘not obese’), all six types of correlations from Table 1 will be considered. Secondly, in order to illustrate the test of whether or not two dependent correlations are equal, we consider an application from Meng et al. (1992) who performed a one-sided test of whether two separate predictor variables, namely the degree of professionalism and the degree of friendliness of an experimenter, have an equal correlation with an outcome variable, namely the experimenter’s expectancy effect, or whether one of the correlations is larger than the other. Finally, in order to illustrate the test of whether or not two independent correlations are equal, we consider a study on the association between two neurological variables, and the same association in a replication study (Forstmann et al., 2010; Boekel et al., 2015). In this test, the null hypothesis, which assumes that the association in the original study and the replication study are equal, reflects a necessary condition in order for the studies to have been executed under the same circumstances and under the same populations. Under the alternative, the associations are unequal, implying that the necessary condition does not hold. Because Bayes factors can be used as measure of support in favor of a null hypothesis, this test allows us to quantify the relative evidence the necessary condition holds, something which is not possible using a significance test.

The paper is organized as follows. In the following section, the methodological background of the Bayesian testing paradigm of correlations is discussed, followed by a section discussing the functionality of the graphical user interface of the module. The next section illustrates how to apply the new module when testing whether each of the six different types of correlations in Table 1 are equal to zero by considering different measurement levels of BMI and physical activity. Subsequently, two illustrative tests of the equality of two dependent correlations (against a one-sided alternative) and the equality of two independent correlations (against a two-sided alternative) are discussed. The paper then provides some methodological considerations, limitations, and extensions (e.g., testing a single partial correlation). The paper ends with concluding remarks.

**Fig. 1:**
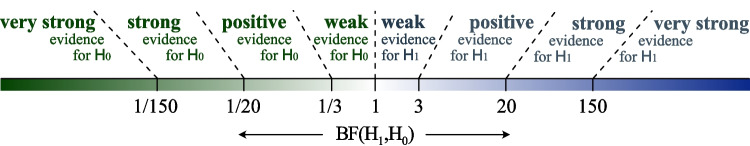
Bayes factor rules of thumb. *Note.* Interpreting the evidence on a continuous scale with qualitative labels (Kass & Raftery, 1995; Mulder et al., 2024)

## Methodological background

We assume the reader has some basic knowledge of Bayesian inference in psychology. If not, we refer the reader to tutorials such as Vandekerckhove et al. (2018) or Hoijtink et al. (2019).

When considering two variables, say, $Y_1$ and $Y_2$, a classical two-sided hypothesis test can be formulated of whether the correlation between these variables, denoted by $\rho$, equals zero or not:

1 $$\begin{aligned} \mathcal {H}_0:\rho =0\text { vs }\mathcal {H}_1:\rho \not =0. \end{aligned}$$

Given a random sample of $Y_1$ and $Y_2$, we can quantify the relative evidence in the data between the two hypotheses $\mathcal {H}_1$ and $\mathcal {H}_0$ using the Bayes factor (Jeffreys, 1961). The Bayes factor is defined as the ratio of the (marginal) likelihoods of the observed data under the two respective hypotheses:

$$ BF(\mathcal {H}_1,\mathcal {H}_0) = \frac{p(Y_1,Y_2\mid \mathcal {H}_1)}{p(Y_1,Y_2\mid \mathcal {H}_0)}. $$

The term ‘marginal likelihood’ refers to the fact that we marginalize the likelihood function based on the relative uncertainty of the parameter values under each hypothesis. The uncertainty of the unknown parameters is reflected in the prior distributions under each hypothesis. Under $\mathcal {H}_0$, the correlation is fixed at zero and therefore no prior needs to be formulated for the correlation coefficient. Under $\mathcal {H}_1$, the correlation can vary in the interval between $-1$ and 1, and therefore a prior needs to be formulated which reflects the relative plausibility of all possible correlation values before observing the data. This prior directly affects the marginal likelihood under $\mathcal {H}_1$, and thereby, directly affects the Bayes factor. To avoid the need to manually choose the prior for the correlation depending on external, contextual information, the BFpack module uses a uniform prior for all possible correlation values. For a single correlation between two variables, this implies a uniform prior for $\rho$ in the interval $(-1,1)$^1^. The uniform prior when testing a single correlation dates back to Jeffreys (1935). The uniform prior differs from the prior that was considered by Wetzels and Wagenmakers (2012) who used a Cauchy prior for a standardized effect in a regression model (Rouder & Morey, 2012) to construct an implicit prior for a correlation. Unlike the Cauchy prior, a uniform prior does not result in unrealistically large probability mass around extreme correlation values near $-1$ and 1 (see Mulder & Gelissen, 2023), and therefore the resulting Bayes factor gives a more realistic quantification of the relative evidence between the hypotheses. For the common nuisance parameters under the two hypotheses, noninformative Jeffreys priors are used. Because of this choice of the priors under the respective hypotheses, the Bayes factor can be computed using a Savage–Dickey density ratio (Dickey, 1971; Mulder & Gelissen, 2023). A Fisher transformation is applied to the correlation to simplify the computation further. Computational details can be found in Appendix A.

The relative evidence in the data between the two hypotheses, as quantified by the Bayes factor, can be interpreted on a continuous scale. For example, a Bayes factor of 10 for $\mathcal {H}_1$ against $\mathcal {H}_0$ would imply that the data were 10 times more likely to be observed under $\mathcal {H}_1$ than under $\mathcal {H}_0$, which implies clear support in favor of $\mathcal {H}_1$. Figure 1 links common qualitative labels to specific ranges of Bayes factors, such as ‘positive evidence’ and ‘strong evidence’ (which date back to Jeffreys, 1961, Appendix B).

When more than two variables are considered, and thus, more than one correlation is tested, the methodology is similar. In this case, a jointly uniform prior is considered for all the correlations in the correlation matrix (e.g., Barnard et al., 2000). This prior assumes that all correlation values are equally likely a priori as long as the implied correlation matrix is positive definite. To ensure positive definiteness, less extreme combinations of correlation values are allowed. For instance, in the case of three variables, the resulting $3\times 3$ correlation matrix contains three correlations, say, $\rho _{12}$, $\rho _{13}$, and $\rho _{32}$, and setting all three correlations equal to $-0.6$ (or more negative values) would not result in a positive definite correlation matrix. Thus, when more variables are included in the analysis, the jointly uniform prior results in marginal priors for the separate correlations that are more concentrated around 0. In the case of three variables, for instance, the jointly uniform prior under the $3\times 3$ correlation matrix results in a marginal prior for each separate correlation having a stretched $beta(\frac{3}{2},\frac{3}{2})$ distribution in the interval $(-1,1)$. This will be illustrated later. In the general case with *P* variables, it has been proven that each marginal prior has a stretched $beta(\frac{P}{2},\frac{P}{2})$ distribution in the interval $(-1,1)$ (Joe, 2006). When data are considered of multiple independent groups, independent jointly uniform priors are specified for the correlation matrices under the independent groups. Finally, note that under a hypothesis with certain constraints on the correlations, e.g., $\rho >0$, the uniform prior approach assumes that all correlation values that satisfy the constraints are equally likely a priori, and correlation values that do not satisfy the constraints would have a prior density of zero.

**Fig. 2:**
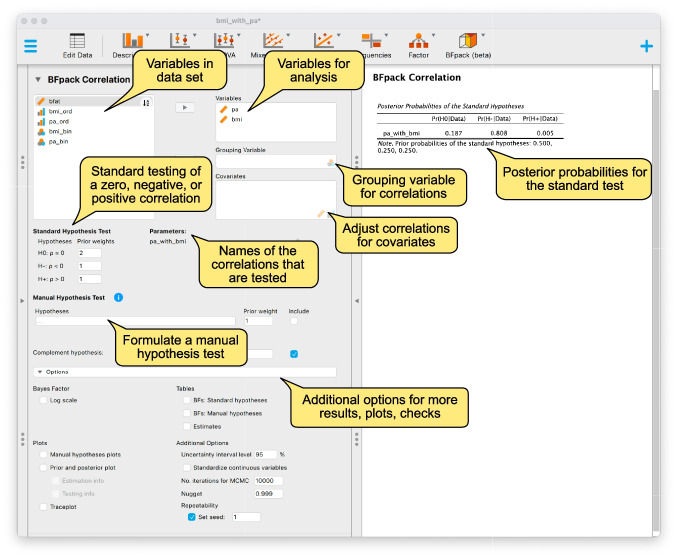
Initial screenshot of the Bayesian correlation test in the BFpack module

In a Bayesian framework, it is also possible to formulate prior probabilities on the hypotheses. The prior probabilities of $\mathcal {H}_0$ and $\mathcal {H}_1$ reflect the plausibility of whether the correlation is zero or whether the correlation is unequal to zero (where all nonzero correlation values are deemed equally likely) before observing the data. A common default choice is to set equal prior probabilities for $\mathcal {H}_0$ and $\mathcal {H}_1$, i.e., $P(\mathcal {H}_0)=P(\mathcal {H}_1)=\frac{1}{2}$. The prior odds can be updated using the Bayes factor from the previous step to obtain the posterior odds of the hypotheses according to

$$ \frac{P(\mathcal {H}_1\mid Y_1,Y_2)}{P(\mathcal {H}_0\mid Y_1,Y_2)} = BF(\mathcal {H}_1,\mathcal {H}_0) \times \frac{P(\mathcal {H}_1)}{P(\mathcal {H}_0)} $$

This formula follows directly from Bayes’ theorem. By setting equal prior probabilities, the posterior odds are equal to the Bayes factor. The BFpack module allows users to freely choose the prior probabilities for the hypotheses. Of course, one does not need to use prior and posterior probabilities of the hypotheses in a Bayesian test, and solely use the Bayes factor to quantify the evidence.

## Getting started with the JASP module

For this tutorial, we first consider the study of Mestek et al. (2008) where the interest was in the association between physical activity (PA; measured by a pedometer) and body composition (BMI; measured by the body mass index). For this illustration, the data of 44 female undergraduate college students are considered. Because the raw data were not available, we generated a synthetic data set with the exact same sample correlation, which was equal to $\hat{r}=-0.38$. The example data can be retrieved from the dedicated open science framework repository (https://osf.io/6sk87). The ‘.jasp’ files that contain the analyses performed in this manuscript can also be found there.

We first open the data file bmi_with_pa.csv in JASP. The data contain variables named ‘bmi’ and ‘pa’, which refer to BMI and PA as measured on a continuous scale. For illustrative purposes, the variables were also discretized according to an ordinal scale with five categories, and labeled as ‘bmi_ord’ and ‘pa_ord’. For the BMI variable, the five categories (1 to 5) correspond to severely underweight (BMI < 16.5), underweight (16.5 $\le$ BMI < 18.5), normal weight (18.5 $\le$ BMI < 25), overweight (25 $\le$ BMI < 30), and obese (30 $\le$ BMI; Weir & Jan, 2023). For the PA variable (measured in steps), the five categories (1 to 5) correspond to sedentary (PA < 5000), low active (5000 $\le$ PA < 7500), somewhat active (7500 $\le$ PA < 10000), active (10000 $\le$ PA < 12500), and highly active (12500 $\le$ PA; Tudor-Locke et al., 2008). For the dichotomous scale, the variables were further discretized into low to normal weight (BMI < 25) vs. overweight (25 $\le$ BMI), and into low active (PA < 10000) vs. highly active (10000 $\le$ PA). These dichotomous variables are labeled as ‘bmi_bin’ and ‘pa_bin’.

After the data have been loaded, we click on the blue “+” symbol in the top right corner of the JASP window in order to access the module list, and click “BFpack”. Next, an analysis can be started by clicking the “BFpack” icon at the top of the screen to unfold a menu from which we select “Correlation”. The left panel shown in Fig. 2 provides a screenshot of some of the input options. The top left block gives an overview of all variables in the data and their measurement level. Correlation testing for scale, ordinal, and binary variables is supported. By moving certain variables to the box labeled ‘Variables’, the module starts an analysis on the correlations between these variables, whose label can be found in the box ‘Parameters’. For example, when moving the variables ‘bmi’ and ‘pa’, the resulting correlation is labeled as ‘bmi_with_pa’. When a user is interested in comparing correlations across different groups, a (categorical) grouping variable can be moved to the box ‘Grouping Variable’. When one is interested in testing correlations while correcting for certain covariates, these covariates can be moved to the box ‘Covariates’.

In the menu, the box ‘Standard hypothesis test’ displays the hypotheses that are tested by default. For the Bayesian correlation test, BFpack executes a multiple hypothesis test to determine whether each separate correlation is zero, negative, or positive, which is formulated as

$$\begin{aligned} &  \mathcal {H}_0:\rho =0\\ &  \mathcal {H}_{-}:\rho <0\\ &  \mathcal {H}_{+}:\rho >0. \end{aligned}$$

The ‘Prior weights’ next to these hypotheses can be used to construct the prior probabilities of the hypotheses (which sum to 1). By default, the prior weights are set to 2, 1, and 1, respectively, which yield prior probabilities of $\text {Pr}(\mathcal {H}_0)=\frac{1}{2}$, $\text {Pr}(\mathcal {H}_{-})=\frac{1}{4}$, and $\text {Pr}(\mathcal {H}_{+})=\frac{1}{4}$. These probabilities are coherent with the default choice in a standard two-sided test of a null hypothesis against a two-sided alternative, each having equal prior probabilities of $\frac{1}{2}$. In the standard test in BFpack, the prior probabilities of $\frac{1}{2}$ for the two-sided hypothesis is equally divided between the two one-sided hypotheses $\mathcal {H}_{-}$ and $\mathcal {H}_{+}$, each receiving prior probabilities of $\frac{1}{4}$. The motivation for the multiple hypothesis test between a zero, negative, or a positive correlation rather than a standard two-sided hypothesis is that the multiple hypothesis test not only provides evidence for or against the null hypothesis, but also which direction receives the most support from the data.

Besides the ‘standard hypothesis test’, other hypothesis tests can be executed using ‘Manual hypothesis test’. This is a flexible tool for testing different types of hypotheses with competing constraints on the correlations of interest. For example, when considering two variables, ‘bmi’ and ‘pa’, resulting in a correlation that is labeled as ‘bmi_with_pa’, and a user would like to test the standard null hypothesis of whether this correlation equals zero, one needs to write ‘bmi_with_pa = 0’. By ticking the box ‘Include’, a manual hypothesis test is executed between this null hypothesis and the complement hypothesis. The complement hypothesis covers the correlation values that are not in agreement with the constraints in the hypothesis that are formulated in the manual hypothesis test. If the null hypothesis, ‘bmi_with_pa = 0’, is the only formulated hypothesis, the complement would correspond to the standard two-sided alternative hypothesis, and thus, a standard two-sided hypothesis test would be executed. Again, the ‘Prior weights’ are used to construct the prior probabilities of the hypotheses in the manual hypothesis (which sum to 1). By default, equal weights of 1 are specified, which implies prior probabilities of $\frac{1}{2}$ in case of the null and two-sided alternative.

In the ‘Options’ menu below, users can specify what output they wish to see in addition to the Bayes factors and the posterior probabilities. Here, we briefly mention certain options that we use in this tutorial paper. By ticking the ‘Estimates’ box, Bayesian estimation of the correlations can be done. The Bayesian estimates are obtained using a certain number of draws from the ‘posterior’, which combines the information in the observed data and the joint uniform prior. The number of draws can be chosen using the option ‘No. iterations’. The default is 10,000 draws resulting in a relative fast analysis. These draws are also (indirectly) used for computing the Bayes factor when testing correlations. By increasing the number of draws, more accurate outcomes are obtained (at the price of longer waiting times for computation). The option ‘Traceplot’ shows the so-called traceplots of all correlations over all iterations. The traceplot is useful to check whether the Bayesian sampling algorithm does not get stuck in extreme correlation values (near $-1$ or 1). This is the case when the traceplots look like hairy caterpillars. If the traceplots show that the algorithm got stuck near $-1$ or 1, however (which might occur in the case of discrete data and very small samples), one can specify a slightly smaller ‘nugget’ than the default value of .999 (e.g., .995 or .99) to avoid the algorithm gets stuck. This will be illustrated later.

## Bayesian two-sided testing of a single correlation

### Testing a Pearson product–moment correlation

**Fig. 3:**
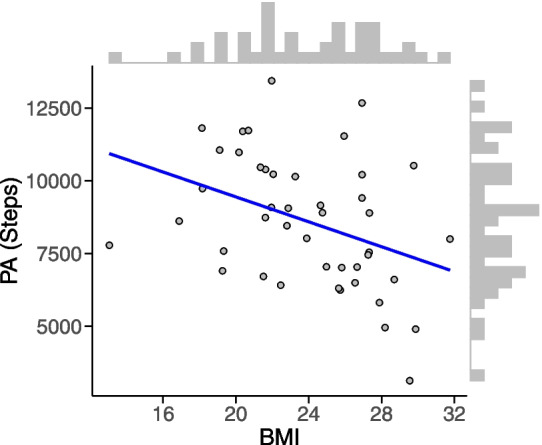
Scatterplot of BMI and PA. *Note.* Scatter plot of the variables BMI versus PA together with their individual histograms and a fitted linear line

The Pearson product–moment correlation quantifies the degree of linear association between the variables that follow an approximate bivariate normal distribution. For this illustration, we consider the variables ‘bmi’ and ‘pa’ which were measured on a continuous scale. The standard ‘Descriptives’ analysis in JASP makes it easy to produce a scatterplot and the associated histograms for the marginal distribution of the two variables. To achieve this, users need to choose ‘Descriptives’ in the top menu, then click ‘Descriptive statistics’, followed by ‘Customizable plots’ and ‘Scatter plot’. Figure 3 shows that the relation between ‘bmi’ and ‘pa’ is negative: unsurprisingly, a lower step count is associated with a higher BMI. However, it is not immediately clear from the figure how much evidence the data provide against the null hypothesis of a zero correlation. By executing a two-sided test using “BFpack”, we can obtain the evidence by quantifying how likely the data would have been under the null (assuming a zero correlation), relative how likely the data would have been under the two-sided alternative hypothesis assuming that any correlation value (unequal to zero) is equally plausible a priori.

To compute the evidence, we perform a Bayesian two-sided test of the product–moment correlation between ‘bmi’ and ‘pa’. We click the “BFpack” icon next to the icons for the other analyses on the ribbon (i.e., along the top of the screen), after which we choose “Correlation” from the list of tests. As illustrated in Fig. 2, assigning the variables ‘bmi’ and ‘pa’ to the ‘Variables’ box immediately initiates two actions: (1) the input panel adds the ‘pa_with_bmi’ correlation underneath ‘Parameters’, which summarizes which model parameters can be tested; and (2) the output panel presents the results of a ‘standard hypothesis test’ of whether the correlation is zero, negative, or positive using prior probabilities for these hypotheses of $\frac{1}{2}$, $\frac{1}{4}$, and $\frac{1}{4}$, respectively.

Before discussing the output, we explain how to specify the common two-sided test of which the correlation is zero or nonzero (which is very similar to the standard test that is executed by “BFpack”). The two-sided test needs to be formulated manually using the ‘manual hypothesis test’. As the manual test indexes the hypotheses using integers, i.e., 1, 2, $\ldots$, we also use this notation here. The motivation is that the traditional null hypothesis of a correlation being zero (which typically has a hypothesis index 0) is not included by default in the manual test. The common two-sided hypothesis test can then be written as

$$\begin{aligned} &  \mathcal {H}_1:\rho =0\\ &  \mathcal {H}_2:\rho \not =0. \end{aligned}$$

As the correlation between ‘bmi’ and ‘pa’ is labeled as ‘pa_with_bmi’, the first hypothesis should be formulated by explicitly writing the following constraint in the text box below ‘Hypotheses’:

$$ \textsf {pa\_with\_bmi = 0} $$

As the two-sided alternative contains all other correlation values that do not satisfy the constraints of the first hypothesis, it is therefore equivalent to the ‘Complement hypothesis’. Therefore, the two-sided alternative does not have to be explicitly formulated when including the complement hypothesis, which is included by default.

To perform the manual test, we need to explicitly tick the ‘Include’ box for the manually formulated hypothesis to the right. Furthermore, we use the typical default choice of equal prior weights of 1 for the two hypotheses, implying equal prior probabilities for $\mathcal {H}_1$ and $\mathcal {H}_2$ of $\frac{1}{2}$. Finally, in the Options menu, we tick the boxes ‘BFs: standard hypothesis test’ and ‘BFs: manual hypothesis test’, which displays the Bayes factors between the hypotheses in the two tests, the ‘Estimates’, which displays the Bayesian mean, median, and lower and upper bound of the 95% credible interval, and the ‘Traceplot’, which illustrates the draws from the posterior over all iterations.

**Fig. 4:**
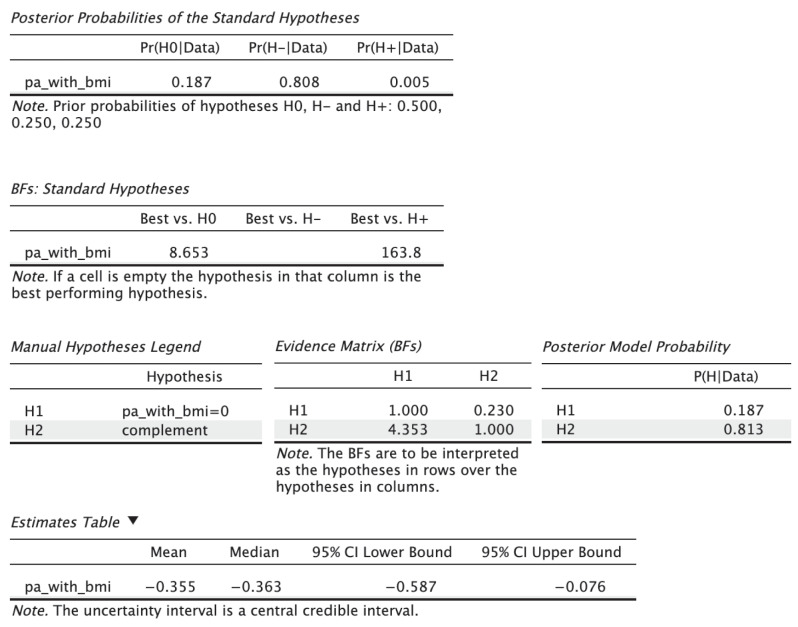
JASP output tables for the product–moment correlation. *Note.* The tables are reorganized so they fit better into the text. In JASP, they will appear underneath each other

**Fig. 5:**
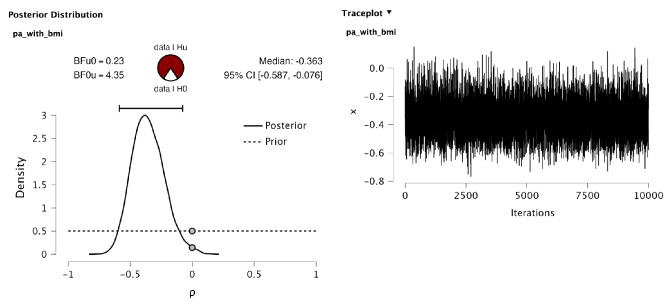
MCMC Plots from JASP. Note. The plot on the left summarizes the prior and posterior distribution of the PA-with-BMI correlation. The plot on the right shows the walk of the posterior MCMC samples for the correlation

In JASP, the output of the analysis is displayed on the right-hand side of the JASP window. The separate tables and plots that we are interested in can be found in Figs. 4 and 5. The first table ‘Posterior probabilities for standard hypothesis test’ displays the posterior probabilities of the hypotheses whether the correlation is zero, negative, or positive given the observed data and the specified prior probabilities. The table shows that the posterior probabilities for the three hypotheses are equal to 0.187, 0.808, and 0.006, respectively. Thus, after observing the data, it is most likely that the correlation is negative with a posterior probability of .808, followed by a posterior probability of .187 for a zero correlation, and finally, the posterior probability for a positive correlation equals .005, suggesting that it is highly unlikely that the correlation between ‘BMI and ‘Pa’ is positive given the 44 paired observations. Next, the table ‘BFs: standard hypothesis test’ gives the Bayes factors for the hypothesis that is most supported against the other two hypothesis. In this case, the hypothesis that received the most support is the hypothesis that assumed the correlation to be negative ($\mathcal {H}_{-}$). Therefore, the table shows the Bayes factor of a negative correlation hypothesis against a zero correlation, which equals 8.653, and the Bayes factor of a negative correlation against a positive correlation, which equals 163.8. These Bayes factors indicate positive evidence for a negative correlation against a zero correlation, and very strong evidence for a negative correlation against a positive correlation.

**Table 2:** Results for the BMI-PA data for different types of correlations

	$BF_{21}$	$P(H_1|Y_1,Y_2)$	$P(H_2|Y_1,Y_2)$	95%-LB	95%-UB	*p* values
Product–moment	4.353	.187	.813	$-0.587$	$-0.076$	.011
Polyserial	1.561	.391	.609	$-0.593$	$-0.010$	.009
Biserial	5.142	.163	.837	$-0.693$	$-0.100$	.009
Polychoric (I)	1.084	.480	.520	$-0.617$	0.030	.026
Polychoric (II)	1.724	.367	.633	$-0.817$	$-0.074$	.003
Tetrachoric	0.761	.568	.432	$-0.708$	0.139	.150

*Note*. BFs for the two-sided alternative ($H_2:\rho \not =0$) against the ‘null’ hypothesis ($H_1:\rho =0$) and their respective posterior probabilities (assuming the hypotheses to be equally plausible a priori) when testing the correlation between physical activity ($Y_1$) and body mass index ($Y_2$) using different measurement levels. 95%-LB and 95%-UB refer to the lower and upper bounds of the 95% credible interval. Classical two-sided *p* values are based on the normal distributions except when testing the product–moment correlation, which is based on a *t* distribution with $n-2$ degrees of freedom

Next, the ‘manual hypotheses legend ’ table shows how the underlying R-package labeled the hypotheses that were specified. The first hypothesis that are specified was the null hypothesis, which is labeled as ‘H1’, and the two-sided alternative (complement) hypothesis is labeled as ‘H2’ in the output.

The ‘evidence matrix ’ contains the Bayes factors between all pairs of hypotheses. The ‘posterior model probability ’ contains the posterior probabilities of the hypotheses under investigation. The ‘estimates table ’ contains the point and interval estimates of the correlations. The Bayes factor for the two-sided test of the product–moment correlation, as well as the posterior probabilities of the two hypotheses, the bounds of the 95% credibility interval, and also the two-sided *p* value are also provided in Table 2.

The Bayes factor for the common two-sided hypothesis, labeled ‘H2’, against the ‘null’ hypothesis, labeled as ‘H1’, shows that the data were 4.353 times more likely to be observed under the two-sided alternative hypothesis than under the null hypothesis. This implies moderate support for a nonzero correlation. Using equal prior probabilities, this Bayes factor yields posterior probabilities for $\mathcal {H}_1$ and $\mathcal {H}_2$ of .187 and .813, respectively. Moreover, the table with the estimates under the full model (which is equivalent to $\mathcal {H}_2$) shows a 95% posterior probability that the correlation lies in the interval $(-.587,-.076)$. Thus, if this interval estimate would be used to perform a classical significance-type test, the null hypothesis would be rejected at the traditional significance level of .05 (the same conclusion would be drawn based on the two-sided *p* values of .011). The Bayesian test gives a more nuanced result: Even though the alternative hypothesis receives more support from the data than the null hypothesis, there is still approximately 19% chance that the correlation is zero after observing the data (see Fig. 4 ‘Posterior Model Probability’ table). This provides another illustration of the fact that significance type testing tends to overestimate the evidence against the null hypothesis, which is a reason for recommending a lower significance threshold of .005 when claiming a scientific discovery (Benjamin et al., 2018). Based on these results, the following could be reported:

“The default Bayesian two-sided test of a product–moment correlation showed that the observed data were about 4.353 times more likely to be encountered under the alternative hypothesis, which assumes that all correlation values from $-1$ to 1 are equally plausible a priori, than under the ‘null’ hypothesis, which assumes that the product–moment correlation equals zero. Thus, there is moderate support in favor of a nonzero correlation. If both hypotheses were equally likely a priori, this yields a posterior probability of .813 for the two-sided hypotheses and a posterior probability of .187 for the ‘null’ hypothesis. Although the posterior probability for the two-sided hypothesis is the highest, the posterior probability for the ‘null’ hypothesis is still too large to claim that the correlation is nonzero with sufficient certainty. More data would be needed to reach a more definitive conclusion.”

Finally, Fig. 5 gives more insights into how the output was obtained. The plot on the left shows the common JASP plot of the estimated posterior of the correlation (solid line) and the prior of the correlation (dashed line), as well as their densities at the null value (grey dots). The Bayes factor for the null hypothesis against the (common) two-sided hypothesis in this correlation test is equal to the ratio of these posterior and prior density values. Furthermore, the plot on the right shows the trace plot, which looks like a (nice) hairy caterpillar. This indicates good posterior mixing and computationally reliable outcomes.

### Testing a polyserial correlation

When two variables follow an approximate bivariate normal distribution but one variable is measured indirectly on an ordinal scale with more than two categories, a polyserial correlation can be used to quantify the degree of linear association between the variables. To illustrate a two-sided test of whether a polyserial correlation equals zero or not, we use the original measurements of the physical activity but discretize the BMI measurements such that each observation falls in one of five possible categories: underweight, healthy weight, overweight, obese, severe obese.

To perform the two-sided test of this polyserial correlation, we remove the variables from the previous analysis and place the variables ‘pa’ and ‘bmi_ord’ in the ‘Variables’ box. We specify the null hypothesis by writing the string ‘pa_with_bmi_ord=0’ underneath the ‘manual hypothesis test’, and we tick the box to include this hypothesis in the manual hypothesis test. Again, the complement hypothesis is automatically included and corresponds to the two-sided alternative.

Following the same procedure as described for the product–moment correlation, we find that the resulting Bayes factor is equal to 1.561 in favor of a nonzero polyserial correlation against a zero polyserial correlation (see Table 2 and Fig. 15 in Appendix B). Thus, the discretization of BMI resulted in a loss of evidence against the ‘null’ hypothesis as compared to the outcome of the two-sided test of the product–moment correlation. As the Bayes factor is close to 1, it can be argued that the data contains very little information to distinguish between a zero and a nonzero correlation, which implies that the study offers the absence of evidence. Under the full model, there would be a 95% chance that the true polyserial correlation lies in the interval $(-0.593, -0.010)$. Given the relatively low support for the two-sided hypothesis, the practical usefulness of this interval estimate is limited. Similarly, note that the two-sided *p* values of .009 imply a serious overestimation of the evidence against a correlation of zero.

**Fig. 6:**
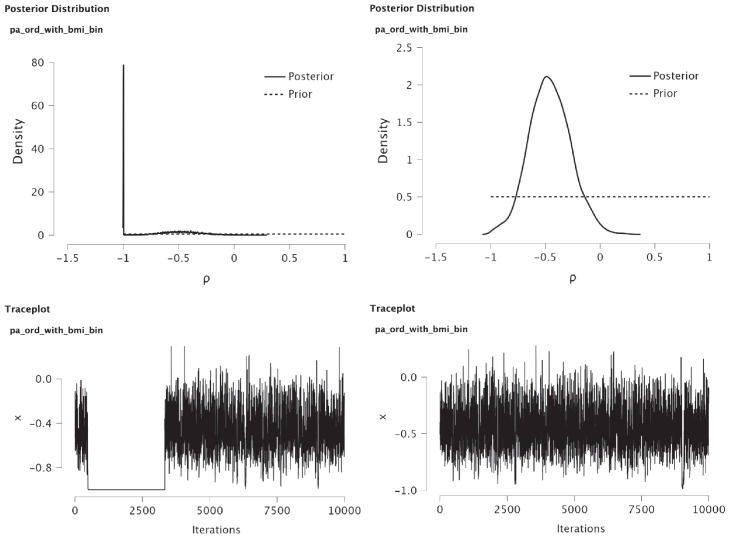
Polychoric correlation (II) MCMC sampling in JASP with different properties. Note. The panels show the MCMC output for the polychoric correlation of PA on an ordinal scale and BMI on a binary scale. The left panel is obtained by leaving the default ‘nugget’ set at .999 leading to non-convergence; the right panel shows proper MCMC output with the ‘nugget’ parameter decreased to .990

### Testing a biserial correlation

The biserial correlation can be used to quantify the degree of linear association between two variables that follow a bivariate normal distribution, where one variable is indirectly measured on a dichotomous scale. To illustrate the two-sided test of a biserial correlation, the original scale of physical activity was used (‘Pa’) and the dichotomized version of BMI, labeled as ‘bmi_bin’, was used (reflecting non-obese vs. obese). Table 2 contains the results of the two-sided test of this biserial correlation (see also Fig. 16 in Appendix B).

The results indicate that the Bayes factor equals 5.142 in favor of the two-sided hypothesis (denoted by $\mathcal {H}_2$) over null hypothesis (denoted by $\mathcal {H}_1$). Interestingly, the evidence against the null hypothesis is relatively strong when compared to the outcome based on the original continuous scale (where a Bayes factor of 4.353 was observed). The posterior probability of the null hypothesis of a zero biserial correlation is also lower now, namely, .163 instead of .187 when using the original scale. Under unrestricted model (having a posterior probability of .837), there is a 95% posterior probability that the biserial correlation lies between $-0.693$ and $-0.100$. Thus, the dichotomization of BMI resulted in a clearer, nonzero relationship between BMI and physical activity.

### Testing a polychoric correlation (I)

The first polychoric correlation can be used when both variables are measured on an ordinal scale, both having more than two categories. To illustrate the corresponding two-sided test, we considered the discretized versions of BMI and physical activity, both of which have five ordinal categories. The variables ‘bmi_ord’ and ‘pa_ord’ are used for the two-sided test. Table 2 shows the results (see also the JASP output in Fig. 17 in Appendix B).

The resulting Bayes factor for the two-sided alternative (denoted by $\mathcal {H}_2)$ against the null (denoted by $\mathcal {H}_1$) is very close to 1, which indicates no clear preference towards either $\mathcal {H}_1$ or $\mathcal {H}_2$. The corresponding posterior probabilities of $\mathcal {H}_1$ and $\mathcal {H}_2$ are also relatively close to each other with .48 and .52, respectively. In this case, the null value is also contained in the 95% credible interval of the polychoric correlation under the full model, which lies between $-0.617$ and 0.030. Using this interval for a two-sided significance test however, it would be unclear whether this nonsignificant result should be interpreted as evidence in favor of a zero correlation or absence of evidence for any of the two hypotheses (Altman & Bland, 1995). Based on the Bayesian test on the other hand, it is clear that the study offers absence of evidence, and that more data are needed in order to reach a stronger conclusion.

### Testing a polychoric correlation (II)

The second polychoric correlation can be used when one variable has a dichotomous scale and the other variable has an ordinal scale. To illustrate the two-sided test, the binary version of BMI, ‘bmi_bin’, and the ordinal version of physical activity, ‘pa_ord’, will be used. After running the Bayesian analysis, the traceplot (i.e., the sequence of posterior samples obtained using Markov chain Monte Carlo techniques) shows that the posterior sampler for the polyserial correlation did not reach convergence, that is, the plot does not resemble a “fat hairy caterpillar” and gets ‘stuck’ around values of $-1$ (see Fig. 6, left panel). JASP also prints a warning if it detects non-convergence indicated by an R-hat statistic that is larger than 1.05 (Gelman et al. 2013, Chapter 11.5).

To avoid this numerical problem, a slightly smaller value of the ‘Nugget scale’ of .990 was used in the options menu. This induces a slight rescaling of the posterior draws of the correlations to avoid the sampler getting stuck in the extremes (we come back to this option at the end of the paper). The resulting traceplot now takes on the desired shape of a “fat hairy caterpillar” (Fig. 6, right panel). The JASP output of the test is printed in Fig. 18 (Appendix B).

The Bayes factor of 1.724 indicates only weak evidence for a nonzero polychoric correlation ($\mathcal {H}_2$; see Table 2). The posterior probabilities of .633 and .367 for $\mathcal {H}_2$ and $\mathcal {H}_1$, respectively, tell a similar story. Under the full model (though the posterior probabilities indicate considerable model uncertainty), there would be 95% chance that the polychoric correlation lies between $-.817$ and $-.074$ (see also the JASP output in Fig. 18 in the appendix).

### Testing a tetrachoric correlation

Finally, the last of these six correlations, the tetrachoric correlation, is useful when the variables are both measured on a binary (dichotomous) scale. A two-sided test of a tetrachoric correlation between the binary versions of BMI and physical activity is used for this illustration by testing the manual hypothesis ‘pa_bin_with_bmi_bin=0’. As the default nugget scale of .999 resulted in the sampler again getting stuck, a slightly smaller nugget scale of .995 was used. Table 2 contains the results of the tetrachoric correlation test (see also the JASP output in Fig. 19 in Appendix B).

For the tetrachoric correlation test, the data were slightly more likely to be observed under the ‘null’ hypothesis ($H_1$) than under the alternative hypothesis ($H_2$), as indicated by a Bayes factor of $BF_{21}=0.761$. As it is close to 1, this again shows the absence of evidence. Also note that the 95% credible interval with upper and lower bounds of $-.708$ and .139 contains 0.

## Bayesian testing of two correlations

Another common testing problem in applied statistical practice concerns the two-sided test of whether or not two correlations are equal. We refer to dependent correlations when the variables are measured within the same population. The correlations are then modeled using the same correlation matrix. Dependent correlations can be *overlapping* when they share a common variable or *nonoverlapping* when they do not share a common variable. When the correlations belong to variable pairs that are measured in distinct independent populations, we speak of independent correlations. Different tests are required for these different pairs of correlations. The BFpack module automatically uses the correct Bayesian test depending on the nature of the relationship between the correlations. In this section, we illustrate a test of dependent overlapping correlations and a test of independent correlations.

### Testing dependent overlapping correlations

When one is interested in whether or not two variables, say $X_1$ and $X_2$, have the same predictive validity for another variable, say *Y*, a test of dependent overlapping correlations can be used. Such an example was discussed by Meng et al. (1992) who considered a one-sided test of whether the association between the degree of professionalism ($X_1$) and experimenter’s expectancy effect (*Y*) was greater than the association between the degree of friendliness ($X_2$) and the so-called experimenter’s expectancy effect (*Y*):

$$\begin{aligned} &  \mathcal {H}_0: \rho _{X_1Y} = \rho _{X_2Y}\\ &  \mathcal {H}_1: \rho _{X_1Y} > \rho _{X_2Y}. \end{aligned}$$

Based on a sample of size $N=15$, the authors reported a classical one-sided *p* values of .047. Using the traditional significance level of .05, the null hypothesis would have been rejected in favor of the one-sided alternative.

Because the raw data are not available, we used the reported (product–moment) correlations ($r_{X_1Y}\!=\!.63$, $r_{X_2Y}\!=\!-.03$, and $r_{X_2Y}=-.19$) to generate an example data set with the identical sample correlations. We load the data, which can be found in the OSF repository (https://osf.io/6sk87), in JASP (named ‘data_meng.csv’).

To perform the one-sided Bayesian test, we place all three variables ‘X1’, ‘X2’, and ‘Y’ in the ‘Variables’ box; immediately, the corresponding parameter names appear underneath the heading ‘Parameters’. In the manual hypothesis test box, we write the constraint of the ‘null’ hypothesis as

$$\begin{aligned} \textsf {Y\_with\_X1 = Y\_with\_X2}. \end{aligned}$$

For the alternative hypothesis, we add another input line by clicking on the green “+” button and subsequently write

$$\begin{aligned} \textsf {Y\_with\_X1 > Y\_with\_X2} \end{aligned}$$

For this illustration, we set equal prior weights of 1 for the two hypotheses, and we exclude the complement hypothesis. Note that the complement hypothesis would encompass the correlation values that do not satisfy the constraints of both hypotheses, which is the parameter region where $\rho _{X_1Y}$ would be smaller than $\rho _{X_2Y}$.

**Fig. 7:**
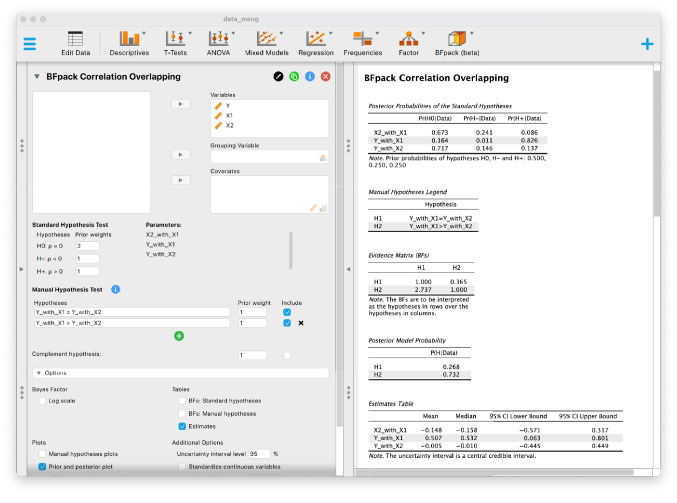
JASP screenshot for dependent overlapping correlations. *Note.* The data example is from Meng et al. (1992)

**Fig. 8:**
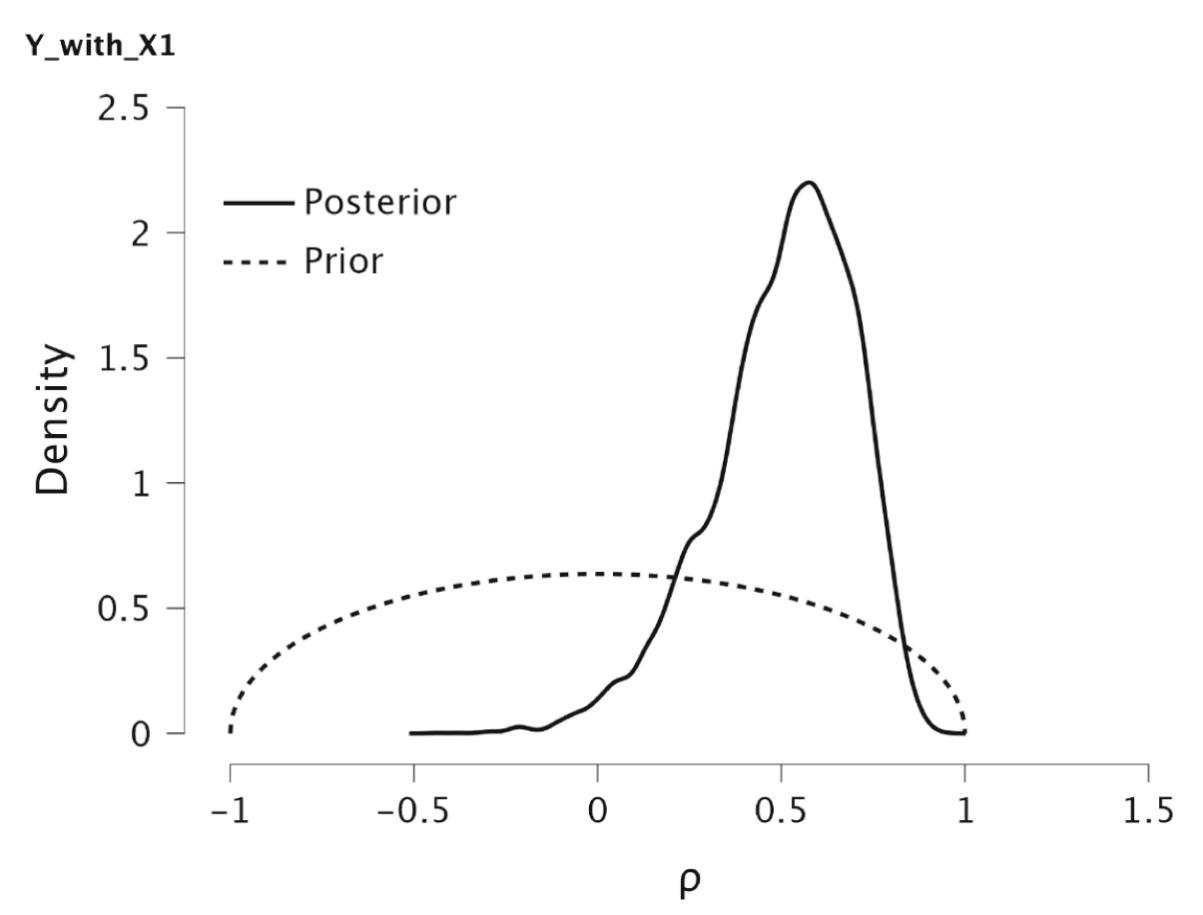
Prior and posterior of the correlation between Y and X1 from the Meng example in JASP. *Note.* The prior of the correlation, $\rho$, corresponds to a stretched $beta(\frac{3}{2},\frac{3}{2})$ prior in the interval $(-1,1)$ which is somewhat concentrated around 0

**Fig. 9:**
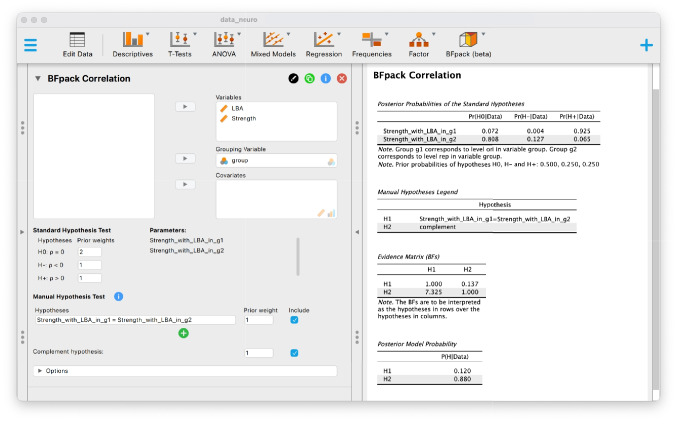
JASP screenshot for independent correlations. *Note.* The data example is comparing the correlation from Forstmann et al. (2010) and the correlation from its replication attempt in Keuken et al. (2017)

From the JASP output, we can see the posterior probabilities for the two hypotheses, and the Bayesian estimates (having checked the ‘Estimates’ box) under the full (unconstrained) model (see Fig. 7). As can be seen, the data were 2.737 times more likely to be observed under the alternative hypothesis ($\mathcal {H}_2$) than under the ‘null’ hypothesis ($\mathcal {H}_1$). This is also reflected in the posterior probabilities of .732 and .268 for $\mathcal {H}_2$ and $\mathcal {H}_1$, respectively. There is only very mild evidence for the one-sided alternative against the null hypothesis. Thus, the result of the Bayesian hypothesis test suggests that the evidence against the null as exemplified by the one-sided *p* values of .047, which would be significant given a significance level of .05, is overestimated. Note that the 95% credible intervals of the two correlations in the Estimates Table in the JASP output also show considerable overlap.

Before discussing the next application, we would like to show how the marginal prior distributions of the separate correlations are affected by the number of variables that are included. Figure 8 shows the marginal prior and posterior of the correlation between X1 and Y. The prior of the correlation corresponds to a stretched $beta(\frac{3}{2},\frac{3}{2})$ prior in the interval $(-1,1)$ which is somewhat concentrated around 0. This is a direct consequence of the uniform prior in the parameter space that results in a positive definite correlation matrix. Including more variables would result in more concentrated marginal priors.

### Independent correlations

When testing the association between two pairs of variables that are measured in different populations, a test of independent correlations is useful. For this illustration, we test whether the correlation between two neurological variables as reported in Forstmann et al. (2010, namely, tract strength between right presupplementary motor area and right striatum, and a linear ballistic accumulator parameter that captures a trade-off between accuracy and speed) was equal to the correlation in the replication attempt reported by Keuken et al. (2017):

$$\begin{aligned} &  \mathcal {H}_0: \rho _{original} = \rho _{replication}\\ &  \mathcal {H}_1: \rho _{original} \not = \rho _{replication}. \end{aligned}$$

The advantage of a Bayesian test for this problem is that we can obtain evidence in favor of the null hypothesis, which assumes that the original study and the replication study have the same correlation between these key neurological variables. This is not possible using classical significance tests, which can only be used for falsifying a null. If evidence were obtained in favor of the null hypothesis, this would imply that the two data sets could be combined for a fixed effects meta-analysis.

We load the data for this analysis, which can be found in the OSF repository (https://osf.io/6sk87), in JASP (named ‘data_neuro.csv’). We move the two variables ‘LBA’ and ‘Strength’ to the Variables box, and we move the ‘group’ variable, which specifies which observations came from the original study (labeled as ‘ori’) and which observations came from the replication (labeled as ‘rep’), to the ‘Grouping Variable’ box. After an initial run, we see the correlation names in the ‘Parameters’ box (see Fig. 9). For these, we can formulate manual hypotheses, namely ‘Strength_with_LBA_in_g1’ and ‘Strength_with_LBA_in_g2’, where the string ‘_in_’ denotes to which group the correlation belongs. Below the first table in the output Posterior probabilities testing standard hypotheses , we then see that the labels ‘g1’ and ‘g2’ refer to the groups ‘ori’ and ‘rep’.

We can execute the two-sided test of independent correlations by specifying the null hypothesis in the manual hypothesis test box by writing:

$$ \textsf {Strength\_with\_LBA\_in\_g1 = Strength\_with\_LBA\_in\_g2} $$

The complement hypothesis corresponds to the two-sided alternative hypothesis. For this illustration, we use equal prior weights for the two hypotheses. The JASP output then shows that the two-sided alternative received about 7.325 more evidence from the data than the null hypothesis (see Fig. 9). Furthermore, given the posterior probability of 0.880 for the alternative hypothesis, it is more likely that different populations or conditions were considered under the original study and the replication study.

## Further considerations, limitations, and extensions

 

- **Default prior specification for the correlations.** The module does not allow manual specification of the joint prior distribution of the correlations, but instead uses a default jointly uniform prior. This prior reflects complete prior ignorance as all correlation values (that result in a positive definite correlation matrix) are assumed to be equally likely a priori, a reasonable default setting for a reference Bayes factor test (e.g., Jeffreys (1961) for testing a single correlation, and Mulder and Gelissen (2023) for testing multiple correlations). Therefore, manually specifying an informative prior based on the anticipated correlation values (e.g., based on the study at hand or the field of study) can be avoided. Finally, note that for testing a single Pearson’s product–moment correlation, JASP has a Bayes factor test implemented based on a stretched beta prior in the interval $(-1, 1)$, which can be used for assessing prior sensitivity.
- **Prior probabilities for hypotheses.** By default, the standard test assumes that a zero correlation has a prior probability of 50%, and a negative or positive correlation both have a prior probability of 25%. For the manually specified hypotheses, equal prior probabilities are assumed by default. Depending on the available external information, however, a user could opt for other choices. For example, if a negative correlation is assumed to be four times more likely a priori than a positive correlation, while a prior probability of 50% is still assumed for a zero correlation, the prior probabilities for a zero, negative, or positive correlation could be set to 50%, 40%, and 10%, respectively. Note that this could be viewed as a weak version of a left one-sided test, as most, but not all, probability mass for a nonzero correlation is placed on the left-sided hypothesis. Furthermore, to assess the robustness of the results, a prior sensitivity analysis can be done using different prior probabilities to assess how robust the conclusions are under different prior assumptions (O’Hagan and Forster, 2004, Chap. 8; Spiegelhalter et al., 2004, Chap. 5). Finally, note that it would be possible to (indirectly) construct informative priors for the correlations via the prior probabilities of the hypotheses. For example, one could divide the parameter space of a correlation into multiple interval hypotheses, e.g., using boundaries of .1, .3, and .5 to distinguish between (very) small, medium, and (very) large correlations. Subsequently, prior probabilities can be specified for the different interval hypotheses to incorporate prior beliefs about whether small, medium, or large correlations are expected a priori. Finally, note here that Bayes factors are unaffected by the prior probabilities of the hypotheses.
- **Checking convergence.** The computational algorithm may encounter convergence issues, especially for small samples and categorical variables. Note that such issues may also be encountered in classical analyses where standard errors may become infinity (e.g., using the hetcor function in the R package polycor). To automatically detect possible convergence issues, the module uses the Gelman–Rubin diagnostic known as R-hat (Gelman et al. 2013, Chapter 11.5). Moreover, checking traceplots is also good practice. In case of convergence problems (i.e., when the traceplots do not look like hairy caterpillars), a slightly smaller nugget scale should be used. As the default is .999, a user can consider using .995, .99, .98, .97, .96, .95, for example. Note that decreasing the nugget scale will result in a slight shrinkage towards zero, implying a slightly more conservative test. For this reason, it is recommended to use smaller nugget scales only to resolve convergence issues.
- **Small samples**. As the module relies on a normal approximation of the posterior of the Fisher-transformed correlations, the approximation may be less accurate in case of very small samples. Note that such limitations also apply in classical tests of correlations, which use normal or *t* sampling distribution, which are less accurate for smaller samples.
- **Numerical error in result**. As the module uses computational algorithms for obtaining the results, there will always be a slight numerical error in the outcome. The default number of iterations is 10,000. When reporting the result in publications a larger number of iterations can be used of, say, 100,000, to increase the numerical accuracy (at the cost of slightly longer waiting times). Relatedly, it is always good practice to report the seed value to ensure exact replicability of the results.
- **Reporting results.** When using the module for empirical analyses in applied scientific work, we refer the reader to the general guidelines for conducting and reporting Bayesian analyses (Van Doorn et al., 2021). These guidelines discuss the four different stages of a statistical analysis including the planning of an analysis, the execution of the analysis, the interpretation of the results, and the reporting of the results.
- **More advanced hypothesis tests.** Besides the tests that were discussed in this tutorial, other tests can be considered as well. For example, it is possible to test correlations while correcting for certain covariates. The covariates should then be placed in the ‘Covariates’ box (Fig. 2). Appendix C presents an illustration for testing the partial correlation between BMI and physical activity while correcting for body fat percentage. The module also allows Bayes factor testing of more than two correlations via the ‘manual hypothesis test’ box. There are various applications of such tests in the literature where competing equality-constrained hypotheses are tested (e.g.., Preacher, 2006; Steiger, 1980), or when testing competing order-constrained hypotheses (e.g., Mulder & Gelissen, 2023; Mulder, 2016). Illustrating these tests falls outside the scope of the current paper.

## Conclusion

The correlation testing framework in the BFpack module in JASP provides researchers with a new set of tools for Bayesian testing of different types of correlation coefficients. This tutorial aims to inform researchers on how to use this module for the most common testing problems of correlation coefficients. In addition to hypothesis testing, the module can also be used for default Bayesian estimation of correlations using the jointly uniform prior.

Given the flexibility of the module for various types of testing problems and the advantages of a Bayesian approach for this purpose, we hope the module encourages researchers to go beyond classical hypothesis testing of correlation coefficients. Researchers will be able to quantify the relative evidence between the hypotheses of interest, given the observed sample, and test scientific expectations in an easy and statistically sound manner.

## Figures and Tables

**Fig. 10:**
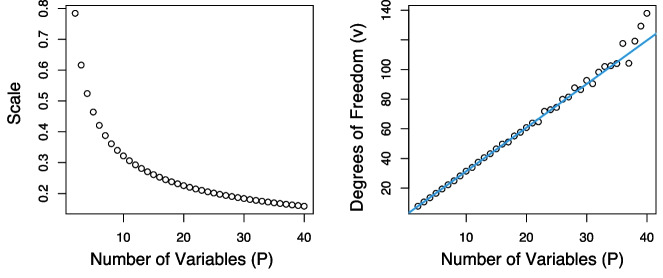
Relationship between number of variables and Student’s *t*-distribution. *Note.* Estimated scale and degrees of freedom of Student’s *t*-distribution of Fisher transformed correlations following a joint uniform prior for a *P*-variate dependent variable. The line in the second panel is an OLS estimate based on the first 20 points

**Fig. 11:**
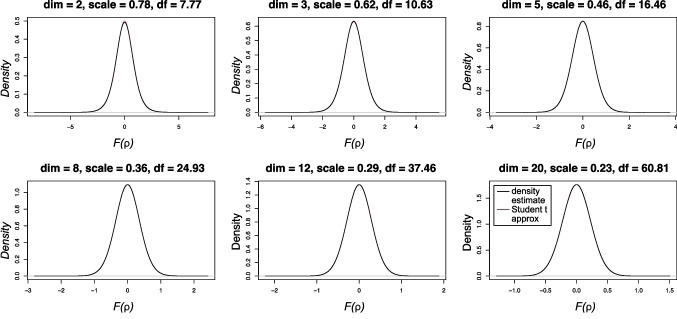
Student’s *t*-approximations. *Note.* Density estimate (*red line*) and Student’s *t*-approximation (*black line*)

**Fig. 12:**
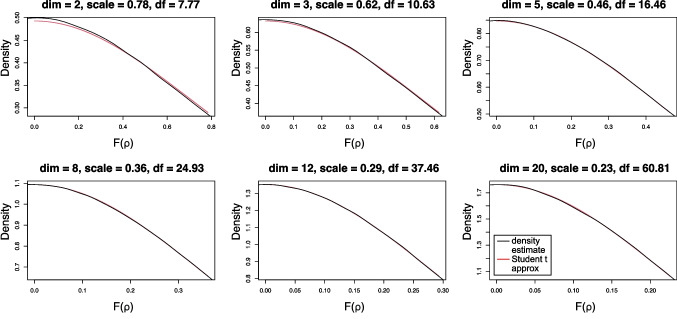
Student’s *t*-approximations. *Note.* Density estimate (*red line*) and Student’s *t*-approximation (*black line*)

**Fig. 13:**
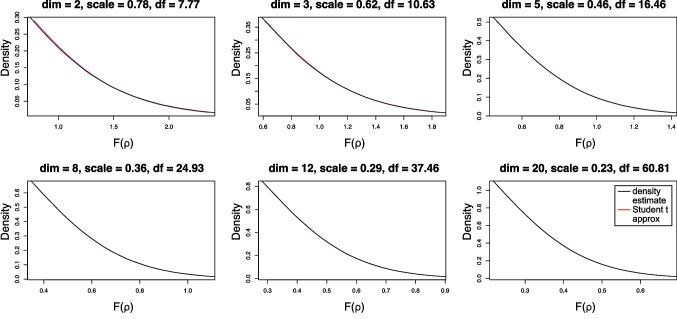
Student’s *t*-approximations. *Note.* Density estimate (*red line*) and Student’s *t*-approximation (*black line*)

**Fig. 14:**
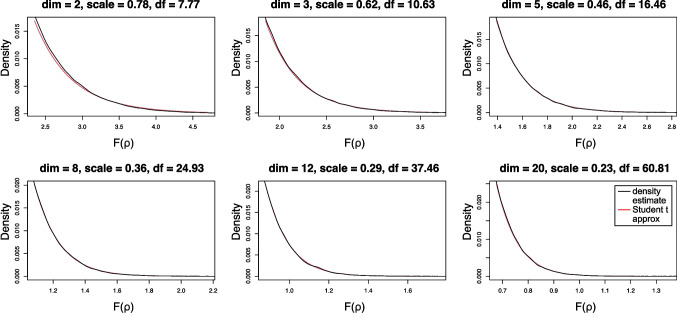
Student’s *t*-approximations. *Note.* Density estimate (*red line*) and Student’s *t*-approximation (*black line*)

**Fig. 15:**
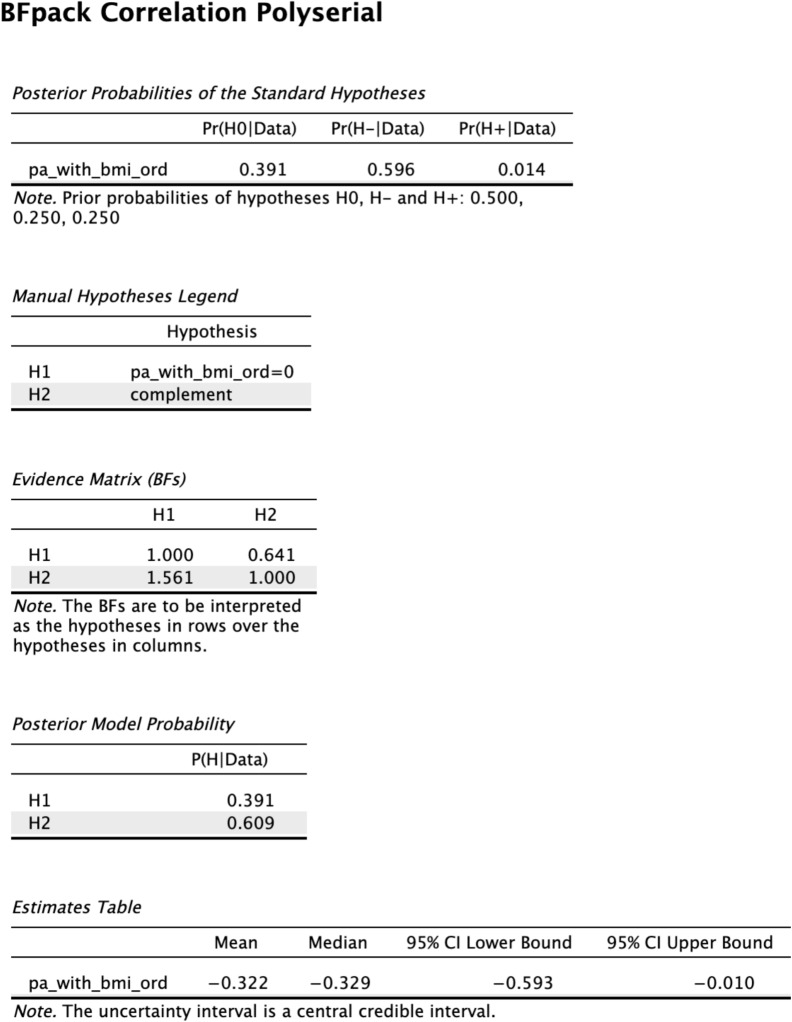
JASP output tables for the polyserial correlation

**Fig. 16:**
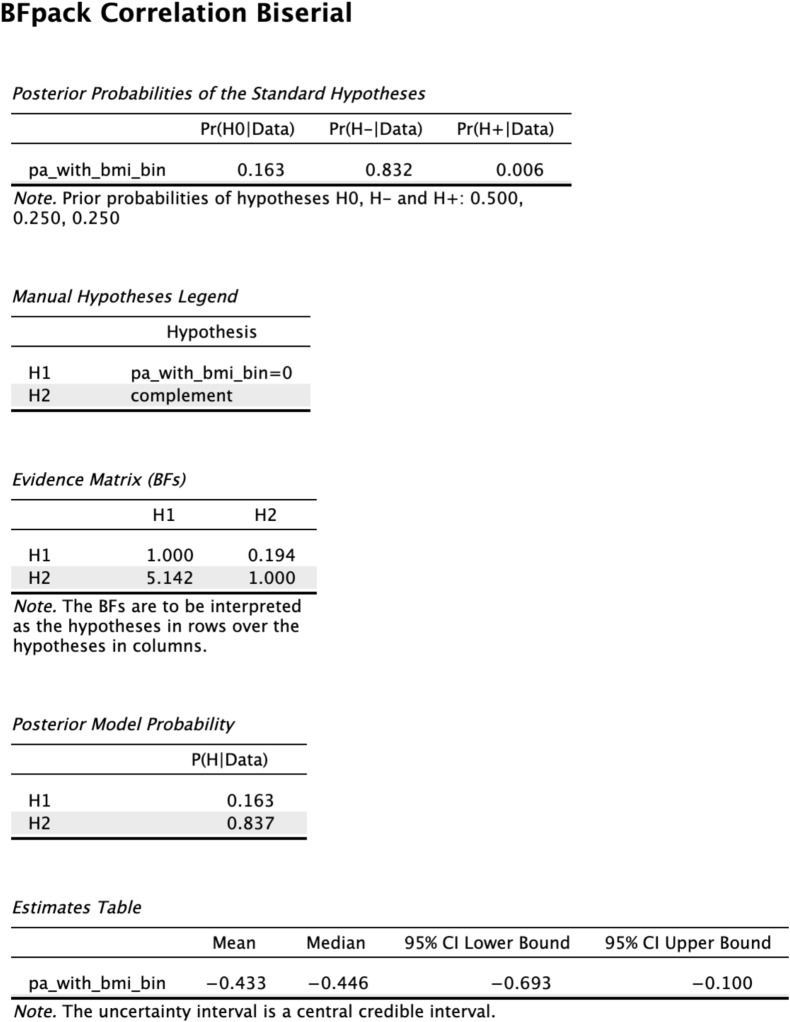
JASP output tables for the biserial correlation

**Fig. 17:**
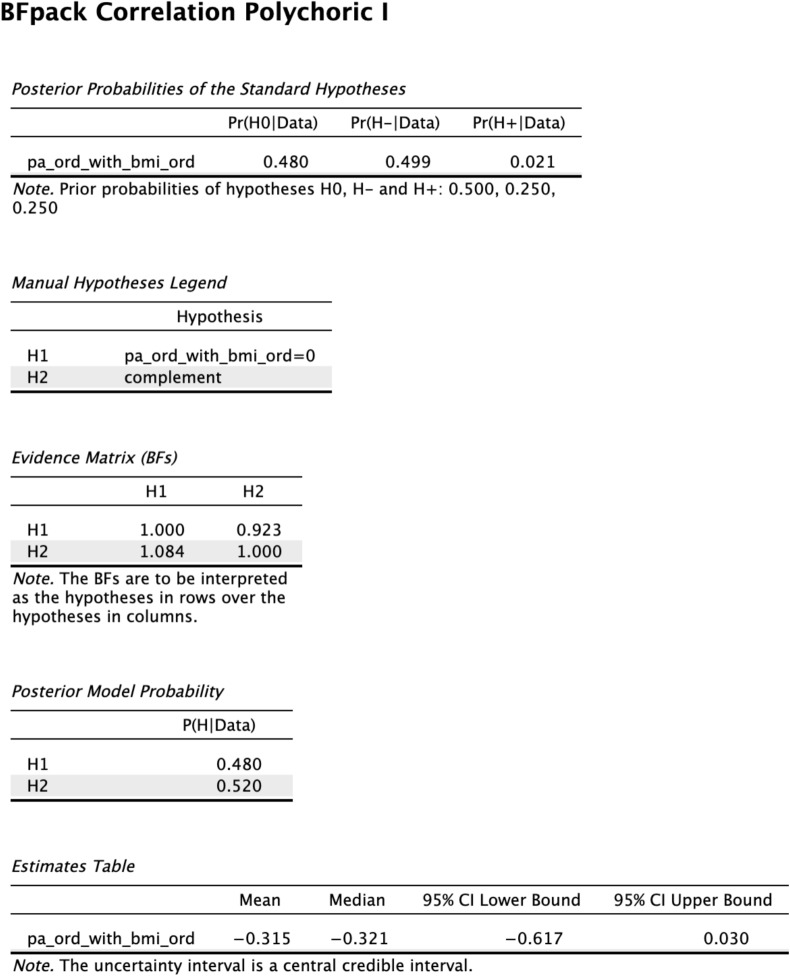
JASP output tables for the polychoric correlation (I)

**Fig. 18:**
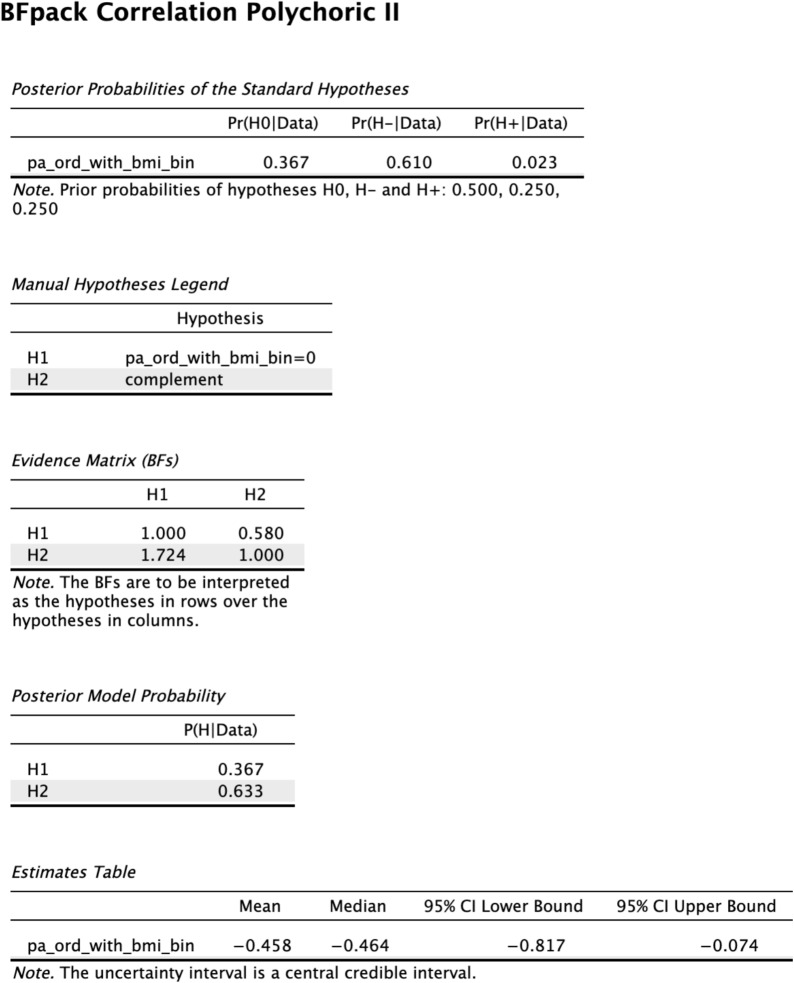
JASP output tables for the polychoric correlation (II)

**Fig. 19:**
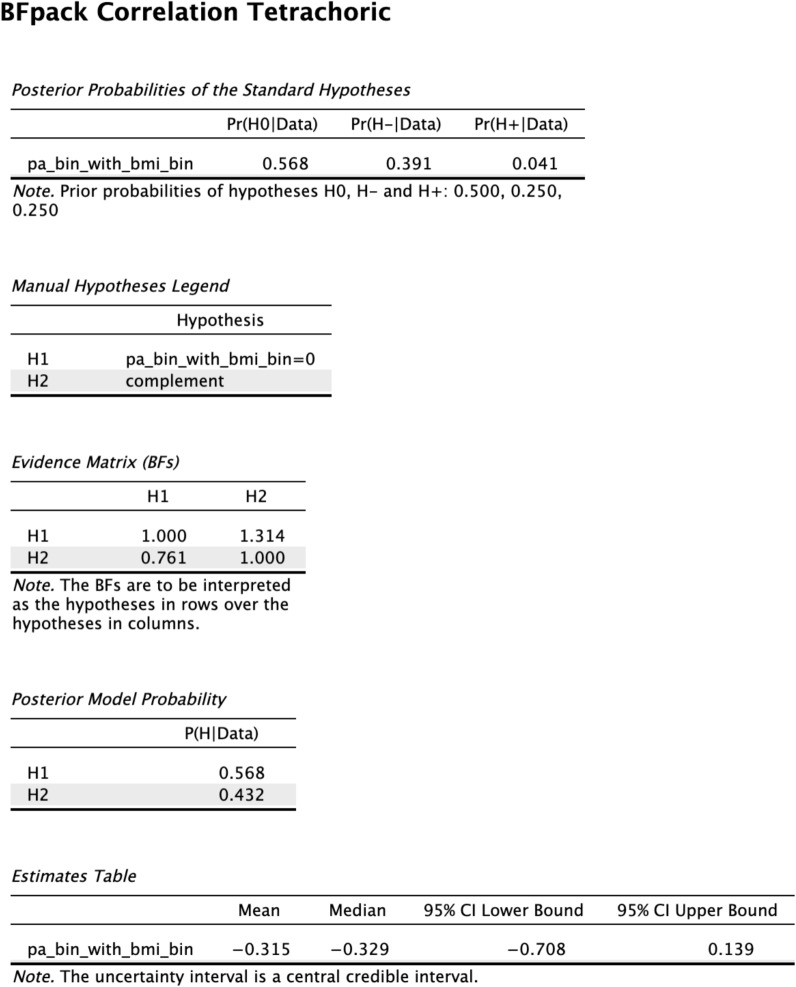
JASP output tables for the tetrachoric correlation

**Fig. 20:**
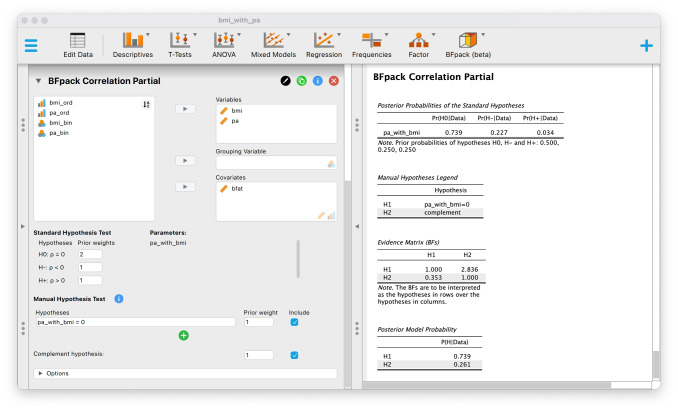
JASP screenshot testing a partial correlation. *Note.* The correlation between body mass index (‘BMI) and physical activity (‘Pa’) corrected for the covariate body fat (‘bfat’)

## Data Availability

The example data sets are available at https://osf.io/6sk87.

## Appendix A Technical details on Bayes factor computation

**Table 3:** Relationship between number of variables and Student’s *t*-distribution

*P*	scale	$\nu$
2	0.784	7.8
3	0.616	10.6
4	0.524	13.5
5	0.464	16.5
6	0.421	19.4
7	0.388	22.2
8	0.361	24.9
9	0.340	28.2
10	0.322	31.4
11	0.306	33.9
12	0.293	37.5
13	0.281	40.5
14	0.270	43.2
15	0.261	46.4
16	0.253	49.7
17	0.245	51.1
18	0.238	55.1
19	0.231	57.6
20	0.226	60.8
21	0.220	63.9
22	0.215	64.7
23	0.210	71.8
24	0.206	72.9
25	0.201	74.4
26	0.197	79.9
27	0.194	81.5
28	0.190	87.6
29	0.187	86.4
30	0.184	92.7
31	0.180	90.4
32	0.178	98.2
33	0.175	102.0
34	0.172	102.5
35	0.170	104.0
36	0.168	117.6
37	0.165	104.2
38	0.163	119.1
39	0.161	129.3
40	0.159	137.9

*Note*. Estimated scale and degrees of freedom of a Student’s *t*-distribution of Fisher transformed correlations following a joint uniform prior for a *P*-dimensional dependent variable.

We write a hypothesis with equality and/or one-sided constraints on a set of correlations as $\mathcal {H}_t:{\textbf {R}}^E_t\varvec{\rho }={\textbf {r}}_t^E~ \& ~{\textbf {R}}^O_t\varvec{\rho }>{\textbf {r}}^O_t$, where ${\textbf {R}}^E_t,~{\textbf {R}}^r_t,~{\textbf {r}}^O_t$, and ${\textbf {r}}^O_t$ are given matrices and vectors that specify the constraints. The prior for the correlations under $H_t$ is uniform under the parameter space where the constraints are satisfied, such that the resulting correlation matrix is positive definite, and zero elsewhere. This prior is a truncated version of the jointly uniform prior under the full unconstrained hypothesis, $\mathcal {H}_u$, denoted by $p_u$. Therefore, the Bayes factor of $\mathcal {H}_t$ against $\mathcal {H}_u$ can be written as an extended Savage-Dickey density ratio (Mulder & Gelissen, 2023):

2 $$\begin{aligned} BF(\mathcal {H}_1,\mathcal {H}_u) = \frac{p_u(\varvec{\rho }^E={\textbf {r}}_t^E|{\textbf {Y}})}{p_u(\varvec{\rho }^E={\textbf {r}}_t^E)} \times \frac{p_u({\textbf {R}}^O_t\varvec{\rho }>{\textbf {r}}^O_t|\varvec{\rho }^E={\textbf {r}}_t^E,{\textbf {Y}})}{p_u({\textbf {R}}^O_t\varvec{\rho }>{\textbf {r}}^O_t|\varvec{\rho }^E={\textbf {r}}_t^E)}, \end{aligned}$$

where $\varvec{\rho }^E={\textbf {R}}^E_t\varvec{\rho }$. Important contributions for this result include Klugkist and Hoijtink (2007), Pericchi et al. (2008), Mulder et al. (2010), and Wetzels et al. (2010).

Equivalently, the Bayes factor can be obtained by computing the four elements in (2) on the Fisher transformed correlations, i.e., $\xi =\frac{1}{2}\log \left( \frac{1+\rho +1}{1-\rho }\right)$. Because the unconstrained posterior of Fisher transformed correlations can be well approximated by a multivariate normal distribution (Mulder & Gelissen, 2023; Mulder, 2016), the two posterior elements in the numerators can be computed directly using the ‘mvtnorm’ package (Genz et al., 2016) when using the fact that the conditional posterior, which is required for computing the posterior probability that the one-sided constraints hold, also follows a normal distribution. Thus, by first obtaining posterior draws of the correlations under the generalized multivariate probit model using the Markov chain Monte Carlo algorithm of (Mulder & Gelissen, 2023), the posterior quantities can be obtained relatively easy.

Furthermore, for a single correlation, it can be shown that the implied prior for a Fisher transformed correlation follows a logistic distribution when starting with a uniform prior for the correlation (e.g., Mulder & van Aert, 2024). Furthermore, a logistic distribution can be well approximated using a Student’s *t*-distribution (O’Brien & Dunson, 2004). To determine the approximated scale parameters and degrees of freedom of the approximate multivariate Student’s *t*-distribution of the Fisher transformed correlations when using a jointly uniform prior for the correlations, numerical estimates were obtained using 1e7 draws from the prior using the sampler of Joe (2006). The Student’s *t*-approximation is obtained using the fit.st() function from the QRM package (Pfaff et al., 2016). The estimated scale and degrees of freedom $\nu$ of the Student’s *t*-distribution for a given dimension *P* of the dependent variable (yielding $P(P-1)/2$ unique correlations) can be found in Table 3 for $P=2$ until $P=40$. Figures 10 plots the estimated scale (left panel) and degrees of freedom (right panel) as a function of the number of variables *P*. The latter also contains a linear OLS estimate of the trend based on the first 20 dimensions (the higher dimensions show more numerical error). The trend can be used for getting more accurate degrees of freedom estimates for higher dimensions. Figures 11-14 show the density estimates (black lines) and the Student’s *t*-approximations (red lines) for six dimensions, $P=2$, 3, 5, 8, 12, 20. Due to the accuracy of the Student’s *t*-distribution to approximate the implied prior of the Fisher transformed correlations when using a jointly uniform prior of the original correlations, this estimated multivariate Student’s *t*-distribution is used. This can be done using the results of Mulder and Gu (2022), who used a Monte Carlo estimate to obtain the conditional probability in case of a multivariate Student’s *t*-distribution.

Hence, using the above approximations of the prior and posterior distribution of the Fisher transformed correlations, the Bayes factor of each constrained hypothesis against the unconstrained hypothesis can be computed relatively easy. Moreover, using the transitivity property of the Bayes factor, it is straightforward to obtain Bayes factor between constrained hypotheses, say, $\mathcal {H}_1$ and $\mathcal {H}_2$, via $BF(\mathcal {H}_1,\mathcal {H}_2) = BF(\mathcal {H}_1,\mathcal {H}_u) / BF(\mathcal {H}_2,\mathcal {H}_u)$.

## Appendix C Testing a partial correlation

The module also allows testing correlation coefficients between variables while correcting for certain covariates. The covariates need to be placed in the ‘Covariates’ box (Figure 20). To illustrate this test, we consider testing the partial correlation between the variables BMI and physical activity, measured on the continuous scale, while correcting for body fat percentage. This variable, labeled as ‘bfat’, is also contained in the data (https://osf.io/6sk87) based on the study of Mestek et al. (2008). Again, a two-sided hypothesis test is considered, which can be written as

$$\begin{aligned} &  \mathcal {H}_0: \rho = 0\\ &  \mathcal {H}_1: \rho \not = 0. \end{aligned}$$

We move the variables ‘bmi’ and ‘pa’ to the ‘Variables’ box and the variable ‘bfat’ to the ‘Covariates’ box. After an initial run, we see the correlation name in the ‘Parameters’ box, which is labeled ‘pa_with_bmi’. Note that the name of the covariate does not appear in the name of the correlation. Next, we write the constraint of the null hypothesis as a manual hypothesis as

$$ \textsf {pa\_with\_pa = 0} $$

We include this hypothesis by clicking ‘Include’. By default the complement hypothesis is included as well, which corresponds to the two-sided alternative in this case. The test is automatically executed (see Fig. 20 for a screenshot). In the ‘Evidence Matrix’, we see that the evidence for the alternative against the null is equal to 0.353, which corresponds to an evidence measure of 2.836 for the null against the alternative. Thus, after correcting for body fat percentage, the direction of the evidence has changed towards the null hypothesis. Recall that evidence for the alternative against the null without accounting for body fat was 4.353. Based on equal prior probabilities for $\mathcal {H}_0$ and $\mathcal {H}_1$, the posterior probabilities are equal to 0.739 and 0.261, respectively. Thus, it is more likely that the partial correlation equals 0. More data would be required to obtain a more decisive outcome.

## Footnotes

1 Strictly speaking the prior excludes the value 0. However, because the parameter lies on a continuous scale, the inclusion or exclusion of one specific value in the interval $(-1,1)$ does not affect the marginal probabilities, and thereby, it does not affect the Bayes factor.

## Code availability

The analysis code is available within JASP (https://jasp-stats.org/download/) for which the source code is at https://github.com/jasp-stats/jaspBfpack.

## Declarations

### Open Practices Statement

No data was collected as part of this paper. The paper was not preregistered.

## References

[CR1] Altman, D. G., & Bland, J. M. (1995). Statistics notes: absence of evidence is not evidence of absence. *Bmj,**311*(7003), 485. 7647644 10.1136/bmj.311.7003.485 PMC2550545

[CR2] Barnard, J., McCulloch, R., & Meng, X.-L. (2000). Modeling covariance matrices in terms of standard deviations and correlations, with application to shrinkage. *Statistica Sinica,**10*, 1281–1311.

[CR3] Benjamin, D. J., Berger, J. O., Johannesson, M., Nosek, B. A., Wagenmakers, E.-J., Berk, R., Bollen, K. A., Brembs, B., Brown, L., Camerer, C., & Cesarini, D. (2018). *Redefine statistical significance. Nature human behaviour,**2*(1), 6–10. 30980045 10.1038/s41562-017-0189-z

[CR4] Berger, J. O., & Delampady, M. (1987). Testing precise hypotheses. *Statistical Science,**2*, 317–335.

[CR5] Boekel, W., Wagenmakers, E.-J., Belay, L., Verhagen, J., Brown, S., & Forstmann, B. U. (2015). A purely confirmatory replication study of structural brain-behavior correlations. *Cortex,**66*, 115–133. 25684445 10.1016/j.cortex.2014.11.019

[CR6] Braeken, J., Mulder, J., & Wood, S. (2015). Relative effects at work: bayes factors for order hypotheses. *Journal of Management,**41*(2), 544–573.

[CR7] Dickey, J. (1971). The weighted likelihood ratio, linear hypotheses on normal location parameters. *The Annals of Statistics,**42*, 204–223.

[CR8] Dienes, Z. (2014). Using bayes to get the most out of non-significant results. *Frontiers in psychology,**5*, 781. 25120503 10.3389/fpsyg.2014.00781 PMC4114196

[CR9] Forstmann, B. U., Anwander, A., Schäfer, A., Neumann, J., Brown, S., Wagenmakers, E.-J., Bogacz, R., & Turner, R. (2010). Cortico-striatal connections predict control over speed and accuracy in perceptual decision making. *Proceedings of the National Academy of Sciences,**107*(36), 15916–15920. 10.1073/pnas.1004932107 PMC2936628 20733082

[CR10] Gelman, A., Carlin, J.B., Stern, H.S., Dunson, D.B., Vehtari, A., & Rubin, D.B. (2013). Bayesian data analysis (3rd ed.). CRC Press: Boca Raton, FL, US. 10.1201/b16018

[CR11] Genz, A., Bretz, F., Miwa, T., Mi, X., Leisch, F., Scheipl, F., ..., Hothorn, T. (2016). R-package ‘mvtnorm’ [Computer software manual]. (R package version 1.14.4 – For new features, see the ‘Changelog’ file (in the package source))

[CR12] Hoijtink, H., Mulder, J., van Lissa, C., & Gu, X. (2019). A tutorial on testing hypotheses using the bayes factor. *Psychological methods,**24*(5), 539. 30742472 10.1037/met0000201

[CR13] JASP Team. (2024). JASP (Version 0.19.0)[Computer software]. Retrieved from https://jasp-stats.org/

[CR14] Jeffreys, H. (1935). Some tests of significance, treated by the theory of probability. *Proceedings of the Cambridge Philosophy Society,**31*, 203–222.

[CR15] Jeffreys, H. (1961). *Theory of probability* (3rd ed.). New York: Oxford University Press.

[CR16] Joe, H. (2006). Generating random correlation matrices based on partial correlations. *Journal of Multivariate Analysis,**97*, 2177–2189.

[CR17] Kass, R. E., & Raftery, A. E. (1995). Bayes factors. *Journal of American Statistical Association,**90*, 773–795.

[CR18] Keuken, M. C., Ly, A., Boekel, W., Wagenmakers, E.-J., Belay, L., Verhagen, J., Brown, S. D., & Forstmann, B. U. (2017). Corrigendum to “A purely confirmatory replication study of structural brain-behavior correlations’’[cortex 66 (2015) 115–133]. *Cortex,**93*, 229–233. 28526139 10.1016/j.cortex.2017.03.007

[CR19] Klugkist, I., & Hoijtink, H. (2007). The bayes factor for inequality and about equality constrained models. *Computational Statistics and Data Analysis,**51*, 6367–6379.

[CR20] Klugkist, I., Laudy, O., & Hoijtink, H. (2005). Inequality constrained analysis of variance: a bayesian approach. *Psychological Methods,**10*, 477–493. 16393001 10.1037/1082-989X.10.4.477

[CR21] Masson, M. E. (2011). A tutorial on a practical bayesian alternative to nullhypothesis significance testing. *Behavior research methods,**43*, 679–690. 21302025 10.3758/s13428-010-0049-5

[CR22] Meng, X.-L., Rosenthal, R., & Rubin, D. B. (1992). Comparing correlated correlation coefficients. *Psychological bulletin,**111*(1), 172.

[CR23] Mestek, M. L., Plaisance, E., & Grandjean, P. (2008). The relationship between pedometer-determined and self-reported physical activity and body composition variables in college-aged men and women. *Journal of American College Health,**57*(1), 39–44. 18682344 10.3200/JACH.57.1.39-44

[CR24] Morey, R. D., Romeijn, J.-W., & Rouder, J. N. (2016). The philosophy of Bayes factors and the quantification of statistical evidence. *Journal of Mathematical Psychology,**72*, 6–18.

[CR25] Mulder, J. (2016). Bayes factors for testing order-constrained hypotheses on correlations. *Journal of Mathematical Psychology,**72*, 104–115.

[CR26] Mulder, J., Friel, N., & Leifeld, P. (2024). Bayesian testing of scientific expectations under exponential random graph models. *Social Networks,**78*, 40–53.

[CR27] Mulder, J., & Gelissen, J. P. (2023). Bayes factor testing of equality and order constraints on measures of association in social research. *Journal of Applied Statistics,**50*(2), 315–351. 36698541 10.1080/02664763.2021.1992360 PMC9870006

[CR28] Mulder, J., & Gu, X. (2022). Bayesian testing of scientific expectations under multivariate normal linear models. *Multivariate Behavioral Research,**57*(5), 767–783. 33827347 10.1080/00273171.2021.1904809

[CR29] Mulder, J., Hoijtink, H., & Klugkist, I. (2010). Equality and inequality constrained multivariate linear models: objective model selection using constrained posterior priors. *Journal of Statistical Planning and Inference,**140*, 887–906.

[CR30] Mulder, J., & Olsson-Collentine, A. (2019). Simple Bayesian testing of scientific expectations in linear regression models. *Behavior Research Methods,**51*, 1117–1130. 30903562 10.3758/s13428-018-01196-9 PMC6538591

[CR31] Mulder, J., & van Aert, R. C. (2024). *Bayesian evidence synthesis: Safely and efficiently combining statistical evidence in meta-analyses*.

[CR32] Mulder, J., Williams, D., Gu, X., Tomarken, A., Böing-Messing, F., Olsson-Collentine, A., Meijerink-Bosman, M., Menke, J., van Aert, R., Fox, J. P., & Hoijtink, H. (2021). Bfpack: flexible bayes factor testing of scientific theories in r. *Journal of Statistical Software,**100*, 1–63.

[CR33] O’Brien, S. M., & Dunson, D. B. (2004). Bayesian multivariate logistic regression. *Biometrics,**60*(3), 739–746. 15339297 10.1111/j.0006-341X.2004.00224.x

[CR34] O’Hagan, A., & Forster, J. (2004) Kendall’s Advanced Theory of Statistics Vol. 2B: Bayesian Inference (2nd ed.) London

[CR35] Pericchi, L. R., Liu, G., & Torres, D. (2008). Objective bayes factors for informative hypotheses: “completing’’ the informative hypothesis and “splitting’’ the bayes factors. In H. Hoijtink, I. Klugkist, & P. A. Boelen (Eds.), *Bayesian evaluation of informative hypotheses* (pp. 131–154). New York: Springer.

[CR36] Pfaff, B., Hofert, M., McNeil, A., Ulmann, S., Pfaff, M. B., McNeil, A. J., Frey, R., Embrechts, P., Rcpp, I., Rcpp, L. (2016). Package ‘qrm’ (version 0.4-13).

[CR37] Preacher, K. J. (2006). Testing complex correlational hypotheses with structural equation models. *Structural equation modeling,**13*(4), 520–543.

[CR38] Rouder, J. N., & Morey, R. D. (2012). Default Bayes factors for model selection in regression. *Multivariate Behavioral Research,**47*(6), 877–903. 26735007 10.1080/00273171.2012.734737

[CR39] Rouder, J. N., Morey, R. D., Speckman, P. L., & Province, J. M. (2012). Default Bayes factors for anova designs. *Journal of Mathematical Psychology.,**56*(5), 356–374.

[CR40] Rouder, J. N., Speckman, P. L., Sun, D., & R. D. M., & Iverson, G. (2009). Bayesian t tests for accepting and rejecting the null hypothesis. *Psychonomic Bulletin & Review,**16*, 225–237. 10.3758/PBR.16.2.225 19293088

[CR41] Sellke, T., Bayarri, M. J., & Berger, J. O. (2001). Calibration of p values for testing precise null hypotheses. *The American Statistician,**55*(1), 62–71.

[CR42] Spiegelhalter, D.J., Abrams, K.R., & Myles, J.P. (2004). Bayesian approaches to clinical trials and health-care evaluation. John Wiley & Sons

[CR43] Steiger, J. H. (1980). Tests for comparing elements of a correlation matrix. *Psychological bulletin,**87*(2), 245.

[CR44] Tudor-Locke, C., Hatano, Y., Pangrazi, R. P., & Kang, M. (2008). Revisiting “how many steps are enough?’’. *Medicine & Science in Sports & Exercise,**40*(7), 537–543. 10.1249/MSS.0b013e31817c7133 10.1249/MSS.0b013e31817c7133 18562971

[CR45] Vandekerckhove, J., Rouder, J. N., & Kruschke, J. K. (2018). Editorial: Bayesian methods for advancing psychological science. *Psychonomic Bulletin & Review,**25*, 1–4. 10.3758/s13423-018-1443-8 29450790

[CR46] Van Lissa, C. J., Gu, X., Mulder, J., Rosseel, Y., Van Zundert, C., & Hoijtink, H. (2021). Teacher’s corner: evaluating informative hypotheses using the bayes factor in structural equation models. *Structural Equation Modeling: A Multidisciplinary Journal,**28*(2), 292–301.

[CR47] Van Doorn, J., van den Bergh, D., Böhm, U., Dablander, F., Derks, K., Draws, T., Etz, A., Evans, N.J., Gronau, Q.F., Haaf, J.M., Hinne, M., Šimon, K., Ly, A., Marsman, M., Matzke, D., Komarlu, A.R., Gupta, N., Sarafoglou, A., Stefan, A., Voelkel, J.G., & Wagenmakers, E.-J. (2021). The JASP guidelines for conducting and reporting a Bayesian analysis . *Psychonomic Bulletin & Review, 28*, 813–826. 10.3758/s13423-020-01798-5 10.3758/s13423-020-01798-5 PMC8219590 33037582

[CR48] Wagenmakers, E.-J. (2007). A practical solution to the pervasive problem of p values. *Psychonomic Bulletin and Review,**14*, 779–804. 18087943 10.3758/bf03194105

[CR49] Wagenmakers, E.-J., Marsman, M., Jamil, T., Ly, A., Verhagen, J., Love, J., Selker, R., Gronau, Q. F., Šmíra, M., Epskamp, S., & Matzke, D. (2018). Bayesian inference for psychology. Part I: theoretical advantages and practical ramifications. *Psychonomic Bulletin & Review,**25*, 35–57. 10.3758/s13423-017-1343-3 PMC5862936 28779455

[CR50] Wasserstein, R. L., & Lazar, N. A. (2016). The asa statement on p-values: context, process, and purpose (Vol. 70) (No. 2). Taylor & Francis.

[CR51] Weir, C. B., & Jan, A. (2023). Bmi classification percentile and cutoff points. Retrieved from https://www.ncbi.nlm.nih.gov/books/ NBK507836/. (Accessed on November 28, 2024)

[CR52] Wetzels, R., Grasman, R. P. P. P., & Wagenmakers, E. J. (2010). An encompassing prior generalization of the savage-dickey density ratio test. *Computational Statistics and Data Analysis,**38*, 666–690.

[CR53] Wetzels, R., & Wagenmakers, E.-J. (2012). A default Bayesian hypothesis test for correlations and partial correlations. *Psychonomic Bulletin & Review,**19*, 1057–1064. 22798023 10.3758/s13423-012-0295-x PMC3505519

